# Validation of loop-mediated isothermal amplification for the detection of *Loa loa* infection in *Chrysops spp* in experimental and natural field conditions

**DOI:** 10.1186/s13071-020-04506-3

**Published:** 2021-01-06

**Authors:** Glory Ngongeh Amambo, Raphael Awah Abong, Fanny Fri Fombad, Abdel Jelil Njouendou, Franck Nietcho, Amuam Andrew Beng, Ritter Manuel, Mathias Eyong Esum, Kebede Deribe, Jerome Fru Cho, Peter Ivo Enyong, Catherine Poole, Achim Hoerauf, Clotilde Carlow, Samuel Wanji

**Affiliations:** 1 Parasites and Vector Research Unit (PAVRU), Department of Microbiology and Parasitology, University of Buea, P.O. Box 63, Buea, Cameroon; 2 Research Foundation in Tropical Diseases and Environment (REFOTDE), P.O. Box 474, Buea, Cameroon; 3 Department of Biomedical science, Faculty of Health Sciences, University of Buea, P.O. Box 63, Buea, Cameroon; 4 Institute of Medical Microbiology, Immunology and Parasitology, University Hospital Bonn, Bonn, Germany; 5 Global Health and Infection Department, Brighton and Sussex Medical School, Brighton, BN1 9PX UK; 6 School of Public Health, Addis Ababa University, Addis Ababa, Ethiopia; 7 New England Biolabs, Ipswich, MA USA; 8 German Center for Infection Research (DZIF), Partner Site Bonn–Cologne, Bonn, Germany

**Keywords:** *Loa loa*, *Chrysops*, RF4-based LAMP, Microscopy, Ivermectin

## Abstract

**Background:**

The mass drug administration of ivermectin for onchocerciasis control has contributed to a significant drop in *Loa loa* microfilaria loads in humans that has, in turn, led to reduction of infection levels in *Chrysops* vectors. Accurate parasite detection is essential for assessing loiasis transmission as it provides a potential alternative or indirect strategy for addressing the problem of co-endemic loiasis and lymphatic filariasis through the Onchocerciasis Elimination Programme and it further reflects the true magnitude of the loiasis problem as excess human mortality has been reported to be associated with the disease. Although microscopy is the gold standard for detecting the infection, the sensitivity of this method is compromised when the intensity of infection is low. The loop-mediated isothermal amplification (LAMP) assay of parasite DNA is an alternative method for detecting infection which offers operational simplicity, rapidity and versatility of visual readout options. The aim of this study was to validate the *Loa loa* LAMP assay for the detection of infected *Chrysops* spp. under experimental and natural field conditions.

**Methods:**

Two sets of 18 flies were fed on volunteers with either a low (< 10 mf/ml) or high (> 30,000mf/ml) microfilarial load. The fed flies were maintained under laboratory conditions for 14 days and then analysed using LAMP for the detection of *L. loa* infection. In addition, a total of 9270 flies were collected from the north-west, east, and south-west regions (SW 1 and 2) of Cameroon using sweep nets and subjected to microscopy (7841 flies) and LAMP (1291 flies plus 138 nulliparous flies) analyses.

**Results:**

The LAMP assay successfully detected parasites in *Chrysops* fed on volunteers with both low and high microfilariaemic loads. Field validation and surveillance studies revealed LAMP-based infection rates ranging from 0.5 to 31.6%, with the lowest levels in SW 2 and the highest infection rates in SW 1. The LAMP assay detected significantly higher infection rates than microscopy in four of the five study sites.

**Conclusion:**

This study demonstrated the potential of LAMP as a simple surveillance tool. It was found to be more sensitive than microscopy for the detection of experimental and natural *L. loa* infections in *Chrysops* vectors. 
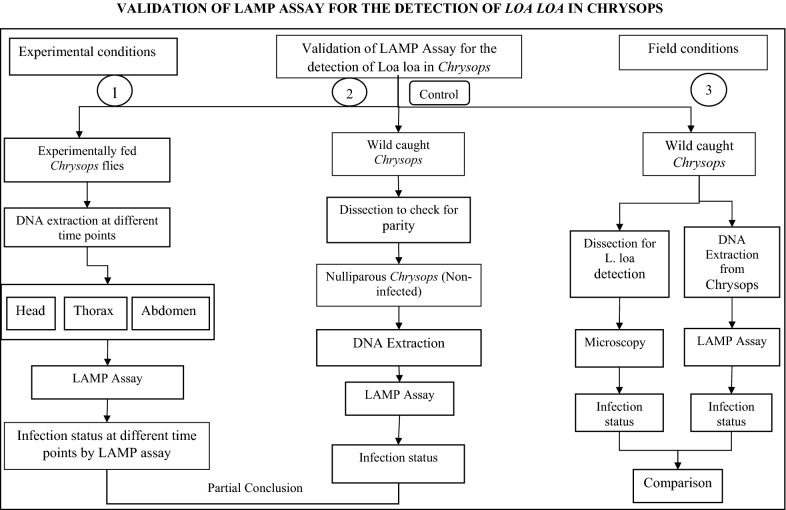

## Background

*Loa loa*, also known as the African eye worm, is a parasitic nematode which causes the neglected tropical disease loiasis. The parasite is transmitted to humans by two main species of tabanid flies of genus *Chrysops*, namely *Chrysops silacea* and *C. dimidiate* [1, 2]. Infection with this filarial nematode is restricted to the rainforest and some savannah areas of Western and Central Africa [3, 4], where an estimated 3 to 13 million people live with the parasite [5]. The burden of disease is highest in Angola, Cameroon, Republic of Congo, Democratic Republic of Congo, Central African Republic, Gabon and Nigeria [4]. Typical reported symptoms include Calabar swellings (transient, localized angioedema) and sub-conjuctival migration of the adult *L. loa* worm [6]. Despite these manifestations, *L. loa* infection has largely been neglected as a public health problem in Africa. *Loa loa*-infected individuals are treated with diethylcarbamazine, which is active against adults and microfilaria(e) (mf) [7] followed by albendazole to eliminate residual mf [8]. The occurrence of Calabar swelling and/or a history of eye worm are used as an indication of infection; however, for definitive diagnosis, detection of mf is required [9]. The monitoring of infection rates in vectors is a rapid and sensitive indicator of the change in community microfilarial load resulting from the distribution of ivermectin, a broad-spectrum anti-parasitic agent [10]. Furthermore, from logistical and ethical perspectives, monitoring infections in the vector offers some advantages over repeated blood examinations of the human population [11]. Specifically, accurate detection of the infection rates in vector populations is essential for assessing transmission, deciding when drug treatments may be stopped and monitoring recrudescence [12]. Several studies have documented reduction of the prevalence and intensity of loiasis in the human population after treatment with ivermectin [13–16]; however, little information is available on the infection rate of the vectors after chemotherapy. In a well-organised control programme, ivermectin would deplete microfilariae in the host, and *Chrysops* flies would tend to take up lower numbers of mf in their blood meals. Thus, accurate diagnostic tests are needed for careful detection of filarial infections in areas where mass drug administration is underway. Currently, the only diagnostic method routinely used for entomological evaluation after chemotherapy is fly dissection using microscopy. While microscopy is a valuable technique, morphological interpretation can be subjective and requires substantial expertise and great effort when large numbers of samples are being processed. In practice, this is not easy for large-scale surveys.

Alternatively, PCR-based molecular assays have been developed that are specific for the detection of *L. loa* mf in blood samples [17–19], which could be optimized for *L. loa* detection in *Chrysops* vectors. However, such assays are time consuming and not generally suitable for use in endemic areas because of the need for highly skilled personnel and high-precision thermocyclers [5]. In addition, the targets of these molecular assays are either present in the genome at a low copy number, which can impact sensitivity, or are not species-specific [20].

Loop-mediated isothermal amplification (LAMP) has emerged as a potential alternative to PCR amplification techniques. LAMP amplifies a target DNA with high specificity, efficiency and rapidity under isothermal conditions [21]. A LAMP assay which targets a highly repetitive DNA target, repeat family 4 (RF4), in *L. loa* has recently been developed [20]. Although this assay represents a major step forward in terms of the search for new diagnostic tools, it has been developed under experimental conditions and, therefore, there is a need to evaluate its performance in the field. The aim of the study reported here was, therefore, to validate the RF4-based LAMP assay as an alternative to dissection for *L. loa* detection in *Chrysops* under experimental and natural field conditions.

## Methods

### Study sites

**Fig. 1:**
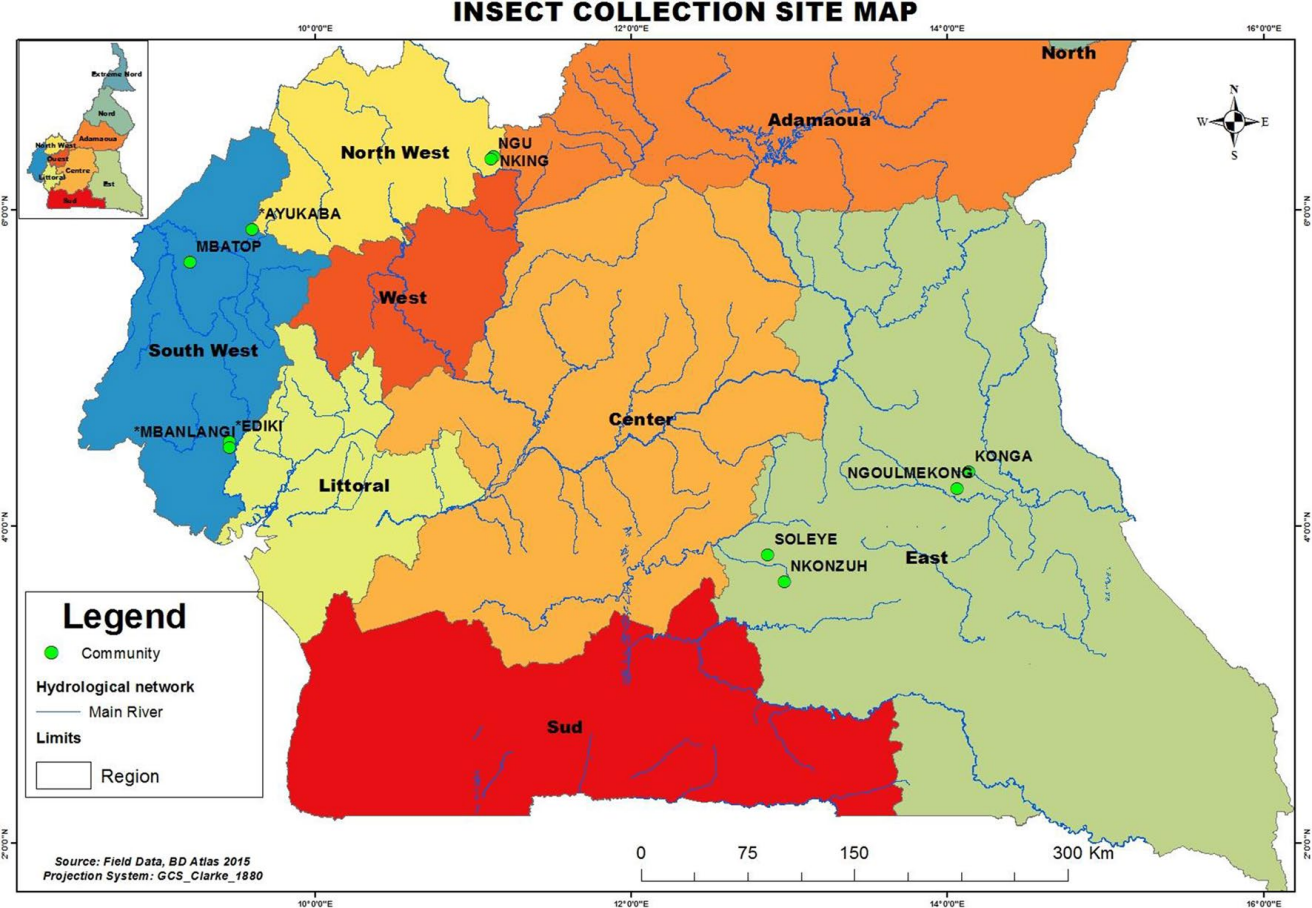
Study sites with *Chrysops* fly collection points shown

Flies were collected from four sites undergoing mass drug administration (MDA) with ivermectin and from a non-MDA site (Batouri Health District) in eastern Cameroon (Fig. 1). The MDA sites included two Community-Directed Treatment with Ivermectin (CDTI) Project sites in the south-west part of Cameroon (SW1 and SW2) and sites in the east (Messamena Health District) and north-west (NW) regions of the country (NWA Health District). The CDTI SW1 site operates within the Mungo and Meme drainage basins and SW2 operates in the Manyu drainage basin [22]. Each of these sites has a different ivermectin MDA treatment profile. SW1 (Kumba Health District) and SW2 (Mamfe Health District) are situated in areas of mild *L. loa* endemicity and have been under CDTI for more than a decade (12–14 years) at the time of this study [23], whereas the eastern and north-western project sites are situated in areas of high *L. loa* endemicity and have been under CDTI for 10 and 9 years, respectively, prior to the study [24, 25]. A CDTI-naïve site (Batouri Health District) in the eastern region of the country was also surveyed. This site is known to be *L. loa* hyper-endemic from a previous study [26].

The climate in the southwest and northwest regions is tropical with two seasons: one wet season of about 9 months, lasting from March to November, and a short dry season, lasting from mid-November to mid-March. The mean annual rainfall in these areas varies from 2500 to 4000 mm. Ambient temperature ranges from 20 ℃ to 40 ℃ depending on the season. The climate in the eastern region is a type A wet equatorial climate [27], with an annual rainfall of 1500–2000 mm and an average temperature of about 24 ℃. This region has four seasons: a long dry season from December to May, a light wet season from May to June, a short dry season from July to October and a heavy wet season from October to November [25].

### Study design

As the aim of the study was to evaluate the performance and suitability of the LAMP assay as a surveillance tool, there were two phases to the study: one involving the use of experimentally infected flies to determine sensitivity and a field phase using wild-caught insects.

### Collection and laboratory maintenance of experimentally fed *Chrysops* flies

*Chrysops* flies were allowed to take blood from consenting microfilaraemic volunteers, then caught using the human landing method using 50-ml Falcon tubes (Corning Inc., Corning, NY, USA). Each tube was prepared to provide suitable conditions for the survival and transport of a single fly, as described previously by Wanji et al. [28]. For the experimental infections, two batches of 18 flies were each fed on either a microfilaraemic volunteer with a low microfilarial load (< 10 mf/ml blood) (Lot 1) or with a high microfilarial load (> 30,000 mf/ml blood) (Lot 2). Once back in the laboratory, the *Chrysops* flies were maintained for up to 14 days to monitor larval development (time for the mf to develop to the third larval stage [L3; infective stage]) in the insectarium. Within this period, the flies were fed daily with a sterile 15% sucrose solution. The temperature of the insectarium was maintained between 23–28 °C and the relative humidity between 79–80 %, as described by Tendongfor et al. [29]. Two flies from each lot were frozen at − 20 ℃ on day 0 (< 7 h post infection [PI]) and on days 1, 4, 6, 7, 10, 11, 12 and 14 PI. At the end of the experiment, the flies were separated into the head, thorax and abdomen, and DNA was extracted from each body part and subjected to the RF4-based LAMP assay for detection of infection.

### Field collection of wild *Chrysops* flies

Insect collections were conducted essentially as described by Duke [30], between 7 a.m. and 6 p.m. from August to October 2014 for a period of 5 days per community. Five trained collectors, dressed in thick clothing that completely covered their body to avoid being bitten by the flies, were stationed near a wood fire. Blood-seeking female flies attracted by the smoke were caught using sweep nets during their attempts to take a blood meal at the different study sites. The number of flies caught per hour was recorded. At the end of each collection session, wild-caught flies were then randomly separated into three groups. The first group served as a control group, as flies from this group were dissected to check for parity; of these 138 nulliparous flies were retained to be further evaluated using the RF4-based LAMP assay. This group served to ascertain the specificity of the assay in the detection of *L. loa* parasite and thereby removed any issues of confounding factors arising from the flies. The remaining two groups were evaluated using either conventional dissection and microscopy or stored in 80% alcohol for DNA extraction followed by LAMP to detect *L. loa* infection.

### Dissection of wild *Chrysops* flies

Wild *Chrysops* were transported in a cold box to the field laboratory and dissected immediately after collection. After a slight jab using a needle tip that knocked out the flies, the flies were dissected in physiological saline (0.9% NaCl) under a dissecting microscope. The head, thorax and abdomen of each fly was separated and placed on slides containing a drop of dissecting medium. The abdomen was teased gently to pull out the ovarioles and spread out to determine the presence (parous) or absence (nulliparous) of follicular relics on the pedicel, as described by Duke [31]. Parous flies were further dissected for the presence or absence of *L. loa* larvae. Larvae were classified into the sausage (L1) stage, larval stage 2 (L2) and L3 following the methods of Duke [32] and Orihel [33]. The infection rates were generated as described by Duke [34] and Noireau et al. [35].

### Purification of DNA from *Chrysops* flies

DNA was extracted using the Zymo Research Genomic DNA Tissue™ MiniPrep Kit (Zymo Research, Irvine, CA, USA) following the manufacturer’s protocol. Briefly, *Chrysops* spp. were crushed individually with the help of sterile micro-pestles in Eppendorf tubes containing 95 μl of water, 95 μl of 2 × digestion buffer and 10 μl Proteinase K solution. The mixtures were incubated at 55 ℃ in a water bath for 1–3 h to denature the nucleases. An 700-μl aliquot of genomic lysis buffer was added to the samples, which were then were mixed thoroughly using a vortex machine followed by a centrifugation step at 10,000 *g* for 1 min to remove insoluble debris. The individual supernatants were carefully transferred to Zymo-spin columns in different collection tubes and then centrifuged at 10,000* g* for 1 min. The columns were removed and inserted into 2-ml collection tubes, and 200 μl of DNA pre-wash buffer was added to the different spin columns in the new collection tubes followed by centrifugation at 10,000* g* for 1 min. Genomic DNA wash buffer (400 μl) was added to the spin columns and centrifuged at 10,000* g* for 1 min. The spin columns were later transferred into clean 1.5-ml Eppendorf tubes. DNA in the spin columns were reconstituted in 200 µl of elution buffer and incubated for 2–5 min at room temperature, followed by a centrifugation step at maximum speed for 30 s. Finally, the DNA was stored at − 20 ℃ until use.

### LAMP assay to detect *L. loa*

The *L. loa* LAMP primers [20] targeting the RF4 that were used for the colorimetric assay were synthesized and purified by high-performance liquid chromatography (Integrated DNA Technologies, Coralville, IA, USA). The primers used and their sequences (5′–3′) were: F3 (TCTTTCYTTTTATCGAGTCGTT); FIP (CGACGTCTTCACAAGGTAAGCC-GTTTAGCCTTGAGTTAGGATC); BIP (GGACACAGAGTAAAATTTACCGCT-CGATTTYCTACTCGTTATTCTTCAA; B3 (AACAGCYTTTGACTCACG); LF (TTAATTAAAGTTCTGCT) and LB (TACAGAGTTGATCAGTAGG).

**Fig. 2:**
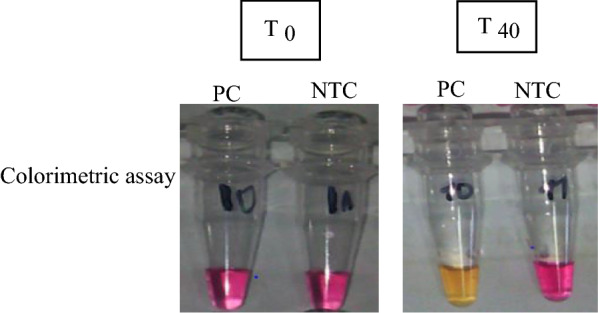
Detection of DNA amplification in the loop-mediated isothermal amplification (LAMP) assay by a change in color. The *Loa loa* primer set was used to amplify genomic *L. loa* DNA using the colorimetric master mix containing phenol red dye and* Bst* 2.0 WarmStart DNA Polymerase (New England Biolabs Inc). Before amplification (*T*_*0*_), reactions are pink. Samples turn yellow if positive for presence of *L. loa* DNA or remain pink if negative, as shown in photographs after a 40-min (*T*_*40*_) amplification.* NTC* non-template control,* PC* positive control

The LAMP reactions contained 1.6 μM of each of primers FIP and BIP, 0.2 μM of each of primers F3 and B3, 0.4 μM of each of primers LF and LB, 12.5 μl of WarmStart Colorimetric LAMP 2× Master Mix (New England Biolabs Inc., Ipswitch, MA, USA) with 2 μl of template DNA or molecular biology grade H_2_0 for non-template controls (NTCs), in a total volume of 25  μl. Reactions were incubated at 61 ℃ for up to 40 min in a GeneAmp® PCR System 9700 Thermal Cycler (Applied Biosystems, Foster City, CA, USA). A detailed description of the method and reaction setup can be found in Additional file 1: Table S1). Samples were considered positive for *L. loa* DNA if an obvious colour change from pink to yellow was observed, while negative samples remained pink (Fig. 2). NTCs were included in each LAMP reaction; the controls never showed signs of amplification.

Due to the high sensitivity of LAMP, precautions were taken to prevent cross-contamination in every experiment. DNA contamination and carry-over of amplified products were prevented by using filter tips at all times, cleaning all work surfaces with a 10% bleach solution before and after each session of work, performing each step of the analysis in separate work areas and minimizing manipulation of the reaction tubes. All tubes are tightly closed and never opened after amplification to avoid contaminating the work area.

### Data processing and analysis

Data were collected and compiled on record sheets and later entered into a template designed in Microsoft Excel 2010 (Microsoft Corp., Redmond, WA, USA). The data were then exported to the SPSS version 20 software package (IBM Corp., Armonk, NY, USA) for subsequent analysis. Contingency tables were used to express the relationship between variables. Fischer’s exact test was used to compare proportions, and all statistical tests were performed at a 5% significance level. The infection rate was determined as the proportion of infected flies to the total number of flies dissected.

## Results

### Detection of *L. loa* infection in experimentally infected* Chrysops* flies using LAMP technology

Equal numbers (*n* = 18) of flies which were fed on a volunteer with either a low or high microfilarial load were analysed using the colorimetric RF4-based LAMP assay. Positive and negative control samples containing either genomic DNA from *L. loa* or molecular biology grade H_2_0, respectively, were included in each assay (Fig. 2). According to the RF4-based LAMP assay, of the 18 flies allowed to engorge on a volunteer with a low parasitaemia level, 16 were positive (88.9%), and of those flies that fed on a volunteer with a high parasitaemia level, 17 (94.4%) scored positive (Table 1).

**Table 1:** *Loa loa* infection rates in experimentally infected *Chrysops* determined by the loop-mediated isothermal amplification assay

Level of parasitaemia	Infection status		Total (*n*)
	Positive,* n* (%)	Negative,* n* (%)	
Low	16 (88.9)	2 (11.1)	18
High	17 (94.4)	1 (5.6)	18
Total	33 (91.7)	3 (8.3)	36

Values in table are presented as a number with the percentage in parentheses

**Fig. 3:**
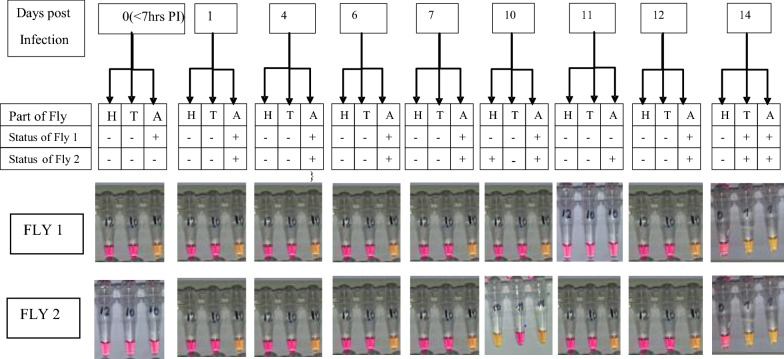
Infection status of flies fed on a microfilaraemic volunteer with a low parasitaemia level. Numbers* 1–14* in boxes at top of figure indicate number of days PI.* H*,* T*,* A* Head, thorax and abdomen of experimentally fed *Chrysops*

**Fig. 4:**
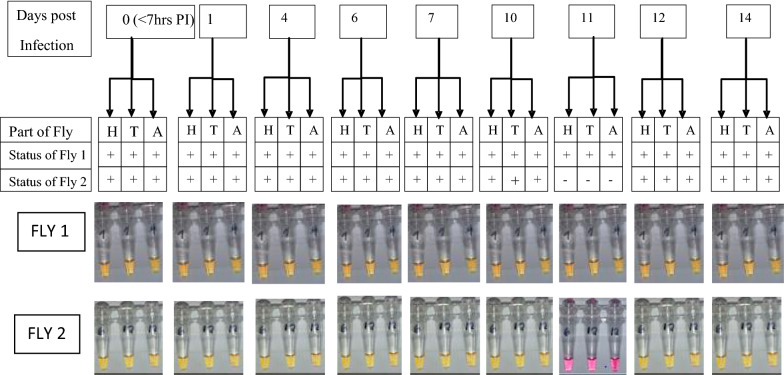
Infection status of flies fed on a microfilaraemic volunteer with a high parasitaemia level. Numbers* 1–14* in boxes at top of figure indicate number of days PI

Comparing the infection status of the two groups of experimentally fed *Chrysops* with respect to different parts of the flies (head, thorax and abdomen) over time, the detection of infection in flies that ingested low mf numbers was limited to the abdomen up to 7 days PI, whereas from day 10 PI onwards, parasites were detected in the head and thorax as well as abdomen (Fig. 3). However, for flies fed on a volunteer with a high parasitaemia level, infection was detected in all the parts of the flies at all-time points examined (Fig. 4).

### Detection of *L. loa* infection in nulliparous *Chrysops* and *Mansonella perstans* samples using the LAMP assay

**Fig. 5:**
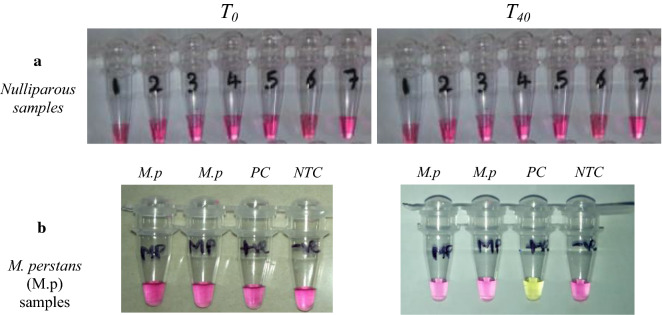
Absence of *L. loa* infection in nulliparous *Chrysops* and *Mansonella perstans* samples. **a** Nulliparous samples, **b** *M. perstans* (*M.P.*) samples compared to NTC and positive control (*PC*). Before amplification (*T*_*0*_), reaction solutions are pink. Samples remained pink after the assay, indicating negativity, as shown on the photographs after a 40-min amplification (*T*_*40*_). Nulliparous *Chrysops* are flies that have never taken a blood meal; as such, samples should remain negative in the assay

We tested DNA from *Mansonella perstans* and a total of 138 nulliparous *Chrysops* flies as negative controls to confirm the specificity of the LAMP assay. No amplification was ever observed in these controls (Fig. 5a, b).

### Detection of *L. loa* infection in wild-caught *Chrysops* using the microscopy method

A total of 7841 wild-caught *Chrysops* from the five study sites were dissected and examined for the presence of *L. loa* infection using microscopy. An overview of the different parasite stages obtained from the flies caught in the different health districts is given in Additional file 2: Table S2. Of these 7841 flies, 257 (3.3%) were found to be infected (Table 2). The highest infection rate, 4.4% (103/2365), was recorded in the non-MDA site (Batouri Health District), followed by 3.5% (17/485) and 3.3% (61/1861) in the NWA and Messamena health districts, respectively. In comparison, 66/2318 (2.8%) and 10/812 (1.2%) of wild-caught flies were *L. loa* positive in the SW 1 and SW 2 CDTI project sites, respectively. Globally, a significant difference (*P* < 0.001) in infection rate was observed between the study sites.

**Table 2:** Natural infection rates of *Chrysops* in the five study sites determined by microscopy and the loop-mediated isothermal amplification assay

Study sites (years of MDA)	Microscopy method		Colorimetric LAMP assay		Fischer’s exact test (*P*-value)
	Total number of wild-caught *Chrysops* flies screened	Number of positive flies (%)	Total number of wild-caught *Chrysops* of flies screened	Number of positive flies (%)	
East MDA (9)	2365	61 (3.3)	183	30 (16.4)	0.000
East non-MDA (0)	1861	103 (4.4)	183	48 (26.2)	0.000
South-west 1 (15)	2318	66 (2.8)	434	137 (31.6)	0.000
South-west 2 (13)	812	10 (1.2)	200	1 (0.5)	0.105
North-west (10)	485	17 (3.5)	291	88 (30.2)	0.000
Total	7841	257 (3.3)	1291	304 (23.5)	0.000

LAMP, Loop-mediated isothermal amplification; MDA , mass drug administration

East MDA, Messamena Health District; East non-MDA, Batouri Health District north-west (NWA Health District); South-west 1, Kumba Health District; South-west 2, Mamfe Health District. See section Study sites for a detailed description

### Detection of *L. loa* Infection in wild-caught *Chrysops* using the LAMP assay

The LAMP assay was performed on DNA extracted from 1291 wild-caught *Chrysops* flies. Of the 1291 flies analysed, 304 were positive, giving an overall infection rate of 23.5% (Table 2). In the non-MDA site, 26.2% (48/183) flies were positive. Similar levels of infection were observed in the north-west (30.2%, 88/291) and SW 1 (31.6%, 138/434) sites. In the eastern MDA site (Messamena Health District), 16.5% (30/183) samples were positive whereas only 0.5% (1/200) of flies scored positive in the SW 2 site.

### Comparison of detection rates of *L. loa* in wild-caught *chrysops* using microscopy and the LAMP assay in various study sites

Overall, the RF4-based colorimetric LAMP assay was found to be significantly more sensitive than microscopy in detecting *L. loa* infection in wild-caught *Chrysops* from the various study sites (*P* < 0.001) (Table 2). An exception was found in the SW 2 CDTI project site where there was no significant difference between the infection rate detected by the two methods (*P* = 0.105).

## Discussion

In Western and Central Africa, co-infection with loiasis and onchocerciasis is a common occurrence [36]. Consequently, entomological evaluation of *L. loa* in the vectors would assist in the development of mathematical models of loiasis transmission and control. While this may not be a solution to reducing the risk of severe adverse effects in the short term, it would provide long-term benefits in terms of the construction of a mathematical model reflecting the epidemiological features of *L. loa* both in the vector and human host, consequently enabling an assessment of the indirect impact of interventions intended to control and eliminate onchocerciasis or lymphatic filariasis and in evaluating the need for further interventions specifically targeting loiasis [37]. Thus, surveying *Chrysops* infection rates in areas where *Onchocerca volvulus* and *L. Loa* are co-endemic is a rapid and an important indicator of transmission that to date depends on microscopic examination*.* However, the detection of *L. loa* larvae in *Chrysops* can be a challenge when parasite densities are low, which is often the case when MDA programmes are ongoing, primarily due to the flies tending to take up lower numbers of mf, thus making microscopic detection difficult. Two studies [5, 38] have described the use of LAMP for *L. loa* using the PCR targets LL3M9 and LLMF72. However, these are not necessarily ideal targets for this platform as LL3M9 contains multiple copies of a simple 6-bp repeat that is conserved in nematodes, and LLMF72 is a single-copy gene that may affect specificity and sensitivity of the assay [20]. Genome filtering for new DNA biomarkers of *L. loa* infection particularly suited to LAMP has resulted in the discovery of several candidates. Of these, RF4, which is highly abundant in the parasite genome, was used to develop a highly sensitive and specific LAMP assay which can detect the DNA equivalent of approximately  1/1600th of a mf [20]. The main goal of the present study was to evaluate the performance of this promising method to detect *L. loa* infection in *Chrysops* spp. using experimentally infected flies and wild-caught flies in natural field conditions.

When LAMP was used to detect *L. loa* in experimentally infected *Chrysops,* the overall infection rate of flies fed on a volunteer with a high parasitaemia level (> 30,000 mf/ml) was 94.4% (17/18 flies) while those fed on a volunteer with a low parasitaemia level (< 10 mf/ml of blood) was 88.9% (16/18). The specificity of the assay was demonstrated using 138 nulliparous flies, none of which scored positive.

In insect vectors, the presence of a peritrophic membrane (PM), which is an extracellular envelope that lines the digestive tract of most insects after blood is ingested, serves as a barrier to infection by pathogens [39, 40] present in blood. It is considered to be the main factor limiting the success rate of microfilarial development following blood infections [41]. Immediately after blood ingestion, the epithelial cells of the posterior midgut secrete the PM, which then envelops the blood. Some mf penetrate the PM before it completely hardens, but the majority die inside the PM. Following ingestion of *L. loa* mf, a period of 7–14 days is required for the parasites to develop into L3 (infective) larvae [42–45]. Development has been reported to take place in the fat body of the head, thorax and—principally—the abdomen of the fly [44, 45]. Interestingly, in *Chrysops* experimentally infected with a low level of infection, LAMP detected parasites as early as day 1 and up to day 7 PI solely in the abdomen. From day 10 onwards, parasites were found throughout the flies. Based on these results, it would thus appear that the infective forms, having migrated to the head, do not remain there until they are offered an opportunity of escaping but that they are capable of freely migrating back to the thorax and the abdomen [44, 46]. In contrast, infection was detected in the head, thorax and abdomen on days 1–14 PI when flies fed on an individual with a high parasitaemia level. The ability of the RF4-based LAMP assay to successfully detect as few as < 10 mf/ml and any developmental stage of the parasite that may be present in the insect hosts suggests the suitability of this method for identification of *Chrysops* with extremely low levels of infection that may be missed using the conventional microscopy method. A high level of sensitivity is particularly important in areas where prolonged administration of ivermectin has led to the drastic reduction of parasite in the host [47].

Microscopy and LAMP assays were used to identify *L. loa*-infected wild-caught *Chrysops* from the different study sites. In general, significantly higher rates of infection were detected using LAMP. The east MDA, east non-MDA, SW 1, and north-west sites recorded the highest infection rates with LAMP (16.4, 26.2, 31.6 and 30.2, respectively) while SW 2 CDTI project site recorded the lowest infection rate of 0.5%.

At the SW 1 CDTI project site, infection rates remained high despite more than a decade of mass treatment with ivermectin. These startling and unsatisfactory observations could be attributed to the persistence of a permanent parasite reservoir. A study by Wanji et al. [22] showed a tepid attitude towards ivermectin intake in the study area. These low adherences in meso- and hyper endemic areas may constitute a permanent transmission of infections in such areas and could be attributed to fear of side effects, as reported by many authors [48, 49]. This finding also tends to confirm findings by Kouam et al. [50] who attributed this stability to the level of exposure of *L. loa* that has not changed after >  10 years of treatment.

LAMP has previously been shown to be more efficient than PCR in detecting *O. volvulus* DNA recovered from black fly material [51]. This better efficacy of the LAMP assy is likely due to insect material containing various biological substances that inhibit the polymerases used in PCR. Indeed, the *Bst* DNA polymerase used in LAMP is more tolerant to PCR inhibitors commonly found in clinical specimens and insects [51–53]. LAMP has also been shown to detect a single mf of *Dirofilaria immitis* in mosquitoes following feeding on infected canine blood [54]. LAMP has other distinct advantages over PCR, including its operational simplicity and isothermal nature. In PCR, thermal cycling is required to denature the template, anneal primers and extend the amplicon. LAMP employs *Bst* DNA polymerase, which provides both strand displacement and target amplification at a single temperature in a simple heat block or water bath at 60–65 ℃ [21] or other portable device with a stable heat source [55]. In addition, LAMP assays have been reported to be significantly cheaper to run than PCR [56]. The rapidity and versatility in readout options also makes the LAMP assay a particularly appealing technology. In its simplest form, as demonstrated here, a clear color change can be easily used in field conditions to indicate amplification of *L. loa* target DNA in infected *Chrysops*. In this study, the colorimetric *L. loa* LAMP assay was used as a rapid qualitative test. However, this LAMP assay can be easily applied as a quantitative approach by adding a fluorescent dye to the colorimetric master mix and incorporating a standard curve to monitor amplification of samples and controls in a real-time PCR machine, as has been described for the *Wuchereria bancrofti* LDR LAMP assay [55]. Alternatively, the change in optical density due to the change of colour from pink to yellow can be monitored using a spectrophotometer, as described by Thi et al. [57].

## Conclusions

This study was designed to validate the LAMP assay for detection of *L. loa* infection rates in *Chrysops spp* in experimental and natural field conditions. The RF4-based LAMP assay as described herein successfully detected *L. loa* parasites in *Chrysops* allowed to feed on individuals with either a low and high parasitaemia level and could also be used to detect infection in wild-caught flies from the different study sites. This molecular assay was considerably more sensitive than the gold standard, microscopy, as it detected a greater number of infected *Chrysops* in four of the five study sites. The assay is also highly specific as no amplification was observed using nulliparous flies or *M. perstans* DNA. The remarkable sensitivity and specificity of the RF4-based LAMP assay and validation of its performance in the field to detect infected *Chrysops* indicate its usefulness as a surveillance tool in global health programmes aimed at achieving elimination of filarial infections.

## Supplementary Information

**Additional file 1: Table S1.** Colorimetric LAMP Protocol for the detection of *L. loa*.

**Additional file 2: Table S2.** The Different parasite stages found in the dissected *Chrysops* flies.

## Data Availability

All data generated or analysed during this study are included within the paper and/or Additional files 1, 2.

## Abbreviations

BIP: Backward inner primer
FIP: Forward inner primer
LAMP: Loop-mediated isothermal amplification
L1, L2, L3: Larval stages one, two, three (infective stage), respectively
MDA: Mass drug administration
mf: Microfilaria(e)
PI: Post infection
RF4: Repeat Family 4
