# Supplementary material for: Validation of loop-mediated isothermal amplification for the detection of Loa loa infection in Chrysops spp in experimental and natural field conditions
Source: Parasit Vectors. 2021 Jan 6;14:19. doi: 10.1186/s13071-020-04506-3 (PMC7788981; doi:10.1186/s13071-020-04506-3)
Supplement: Supplementary file 1 — Additional file 1: Table S1. Colorimetric LAMP Protocol for the detection of L. loa. [file 13071_2020_4506_MOESM1_ESM.docx]

**Colorimetric LAMP Protocol for the detection of *L. loa***

**1. 25X Primer Mixes:**

| ^g^Standard Primers | Volume (μl) | 25X concentration | 1X concenration |  |
| --- | --- | --- | --- | --- |
| 100 μM FIP | 40 | 40 μM | 1.6 μM |  |
| 100 μM F3 | 5 | 5 μM | 0.2 μM |  |
| 100 μM BIP | 40 | 40 μM | 1.6 μM |  |
| 100 μM B3 | 5 | 5 μM | 0.2 μM |  |
| H_2_O | 10 | ---- | ---- |  |
| Total Volume | 100 | ---- | ---- |  |

| ^g^Loop Primers | Volume (μl) | 25X concentration | 1X concentration |  |
| --- | --- | --- | --- | --- |
| 100 μM LF | 10 | 10 μM | 0.4 μM |  |
| 100 μM LB | 10 | 10 μM | 0.4 μM |  |
| H_2_O | 80 | ---- | ---- |  |
| Total Volume | 100 | ---- | ---- |  |

**2. Colorimetric LAMP reactions:**

| Components | Volume (μl) |
| --- | --- |
| 2X Warmstart colorimetric Master mix | 12.5 |
| 25X Standard Primer mix | 1 |
| 25X Loop primer mix | 1 |
| ^i^Substrate DNA | 2 |
| H_2_O | 8.5 |
| Total Volume | 25 |

a. 100 μM primer stocks are prepared in H_2_O to minimize carry over of Tris.

b. LAMP reactions are incubated in a GeneAmp®, PCR System 9700 Applied Biosystems @ 61^o^C for 40 min as described in the Materials and Methods.

c. Substrate DNA can be dissolved in either elution buffer or H_2_O.
